# Biosynthesis of Astaxanthin as a Main Carotenoid in the Heterobasidiomycetous Yeast *Xanthophyllomyces dendrorhous*

**DOI:** 10.3390/jof3030044

**Published:** 2017-07-30

**Authors:** Jose L. Barredo, Carlos García-Estrada, Katarina Kosalkova, Carlos Barreiro

**Affiliations:** 1 CRYSTAL PHARMA S.A.U. Parque Tecnológico de León, C/Nicostrato Vela s/n, 24009 León, Spain; JoseLuis.Barredo@amriglobal.com; 2 INBIOTEC (Instituto de Biotecnología de León), Avda. Real, 1, 24006 León, Spain; cgare@unileon.es (C.G.-E.); kkos@unileon.es (K.K.); 3 Área de Toxicología, Departamento de Ciencias Biomédicas, Universidad de León, Campus de Vegazana, 24071 León, Spain; 4 Área de Microbiología, Departamento de Biología Molecular, Universidad de León, Campus de Ponferrada, Avda, Astorga, s/n, 24400 Ponferrada, Spain

**Keywords:** *Xanthophyllomyces dendrorhous*, *Phaffia rhodozyma*, astaxanthin, carotenoids, carotenes, xanthophylls

## Abstract

Carotenoids are organic lipophilic yellow to orange and reddish pigments of terpenoid nature that are usually composed of eight isoprene units. This group of secondary metabolites includes carotenes and xanthophylls, which can be naturally obtained from photosynthetic organisms, some fungi, and bacteria. One of the microorganisms able to synthesise carotenoids is the heterobasidiomycetous yeast *Xanthophyllomyces dendrorhous*, which represents the teleomorphic state of *Phaffia rhodozyma*, and is mainly used for the production of the xanthophyll astaxanthin. Upgraded knowledge on the biosynthetic pathway of the main carotenoids synthesised by *X. dendrorhous*, the biotechnology-based improvement of astaxanthin production, as well as the current omics approaches available in this yeast are reviewed in depth.

## 1. Introduction: *Xanthophyllomyces dendrorhous* and Carotenoids

The carotenoids group includes tetraterpenoid organic pigments, the majority comprising of eight isoprene units with a C40 skeleton. These lipophilic metabolites are insoluble in water and contain a long polyene central chain of conjugated double bonds that functions as a chromophore (400–500 nanometers being the electromagnetic spectrum where carotenoids absorb maximally) responsible for the characteristic yellow to orange and reddish colours of these compounds [1].

All naturally occurring carotenoids are produced by photosynthetic species (including plants and algae), and by some classes of fungi and non-photosynthetic bacteria [2,3,4,5]. In general, animals are unable to produce their own carotenoids and therefore, the only way to obtain these compounds is from their diet.

Carotenoids can be classified according to the oxygenation degree into carotenes and xanthophylls. Carotenes (e.g., β-carotene, α-carotene or lycopene) are strictly hydrocarbons (non-oxygenated molecules), whereas xanthophylls (e.g., lutein, zeaxanthin, canthaxanthin, or astaxanthin) are oxygenated molecules (oxycarotenoids) with a hydroxy, epoxy, and/or oxo group [6]. These compounds play different roles. Thus, in photosynthesizing species they are associated with the light harvesting complexes acting as accessory light-harvesting pigments, effectively extending the range of light absorbed by the photosynthetic apparatus. In those organisms, carotenoids also play a photoprotective role by quenching triplet state chlorophyll molecules and scavenging singlet oxygen and other toxic oxygen species formed within the chloroplast, and in the case of zeaxanthin, by dissipating harmful excess excitation energy under stress conditions [7,8]. Besides, carotenoids provide organisms of bright yellow, red, or orange and their main function in all non-photosynthetic organisms seems to be (photo) protection. They are known to be very efficient physical and chemical quenchers of singlet oxygen (^1^O_2_), as well as potent scavengers of other reactive oxygen species, playing an important role as antioxidants [9,10]. Carotenoids are also important precursors of retinol (vitamin A precursors) [11].

More than 700 types of carotenoids have been found from natural sources so far [5,12]. The carotenoids market in 2019/2020 is supposed to reach $1.5–1.8 billion with a compound annual growth rate of 3.9% [13,14] and due to the extensive commercial and industrial uses of carotenoids (mainly lutein and astaxanthin), the demand of these compounds is high around the world. Therefore, several biological platforms are used for the biotechnological production of natural carotenoids for their use in food and feed, cosmetics, and the chemical and pharmaceutical industries [15].

One of these platforms is the red/pink-pigmented heterobasidiomycetous yeast *Xanthophyllomyces dendrorhous* (the teleomorphic state of *Phaffia rhodozyma*), which was isolated in the late 1960s from tree-exudates in Japan and Alaska [16] and naturally produces and accumulates the xanthophyll astaxanthin [17]. This yeast can be considered as a cell-factory for the production of industrially valuable carotenoids [18], since the genome of *X. dendrorhous* CBS6938 has been sequenced [19] and biotechnology tools for the genetic manipulations of this microorganism are available [20,21,22,23]. In addition, this yeast does not require light for accumulation of astaxanthin, is able to metabolise many kinds of saccharides under both aerobic and anaerobic conditions, and reproduces at relatively high growth rates [24]. Besides, its approval as a colour stabiliser for fish feed supplementation by FDA (U.S. Food and Drug Administration) supports the natural production as an interesting methodology (https://www.accessdata.fda.gov/scripts/cdrh/cfdocs/cfcfr/CFRSearch.cfm?fr=73.355).

**Figure 1:**
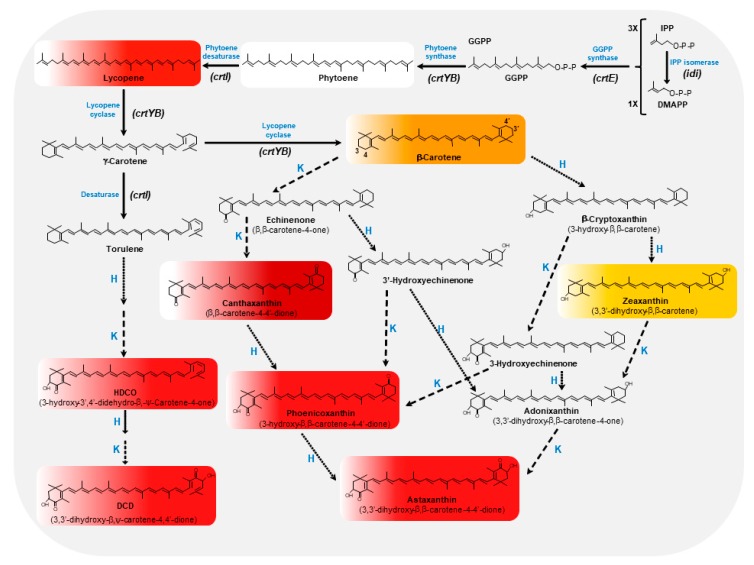
Astaxanthin biosynthetic pathway in *X. dendrorhous*. Initially, a molecule of dimethylallyl pyrophosphate (DMAPP) and three molecules of isopentenyl pyrophosphate (IPP) are combined to obtain geranylgeranyl pyrophosphate (GGPP) by means of the GGPP synthase. Secondly, two molecules of GGPP are coupled by the phytoene synthase (*crtYB* gene) to reach phytoene. The phytoene desaturase(*crtI* gene) introduces four double bonds in the molecule of phytoene to to obtain lycopene. Then, the lycopene cyclase (*crtYB* gene) converts one of the ψ acyclic ends of lycopene as β-ring to form γ-carotene, and subsequently the other to form β-carotene. Xanthophylls bioconversion in *X. dendrorhous* from β-carotene and γ-carotene includes the addition of two 4-keto groups in the molecule of β-carotene by the ketolase (K, discontinuous line) activity, and the inclusion of two 3-hydroxy groups by the hydroxylase (H, continuous dotted line) activity. Both K and H activities are presented in a single enzyme (astaxanthin synthetase; CrtS) encoded by a single gene (*crtS*). The cytochrome P450 reductase encoded by the *crtR* gene is a CrtS helper protein, providing with electrons for substrate oxygenation. The existence of a monocyclic pathway to DCD was also proposed [42]. Main carotenoids detected in *X. dendrorhous* broths are shown inside a rectangle in concordance with their natural colours. This figure has been based on Rodríguez-Sáiz and co-workers [43].

In addition to astaxanthin, *X. dendrorhous* is able to produce several carotenoids, including β-carotene, canthaxanthin, zeaxanthin, and astaxanthin via mevalonate pathway (Figure 1). β-carotene, a red-orange carotenoid, possesses β-rings at both ends and serves as an intermediary molecule for the biosynthesis of astaxanthin [25,26]. This compound is widely used in the food, feed, cosmetic, and pharmaceutical industries due to its potent colouring traits, antioxidant properties, and provitamin A activity, since it appears to be the most important vitamin A precursor for vertebrate animal species [27]. Besides, β-carotene adds colour to beverages, dairy products, confectionery, and many other commodities, and together with astaxanthin, they are the most important carotenoids from a commercial point of view. The orange-red pigment canthaxanthin (β,β-carotene-4,4′-dione) is a xanthophyll with antioxidant properties widely used in aquaculture and poultry farming, providing the characteristic colour to fish flesh, chicken skin, and egg yolk [28,29]. Zeaxanthin (β,β-carotene-3,3′-diol) is a yellow xanthophyll alcohol important for vision, since together with lutein and meso-zeaxanthin, they are present in high concentrations within the oval-shaped macular pigmented area near the centre of the retina of the eye [30,31], thus playing an important protective role against several eye diseases [32]. Astaxanthin (3,3′-dihydroxy-β,β-carotene-4,4′-dione) is a red-orange pigment with a market value ranging from $2500–7000/kg whose global market was valued at US$447 in 2014 and expected to reach a value of US$1.1 billion by 2020 [14,33]. After β-carotene, astaxanthin is the second most important carotenoid, representing about 29% of total carotenoid sales [34]. This pigment has strong antioxidant properties and is used as a feed additive in salmon and trout aquacultures as well as in chicken and quail farming and egg production [6,35,36]. Other properties have been described for astaxanthin, such as beneficial effects in cardiovascular, immune, inflammatory, diabetes, carcinogenic, and neurodegenerative diseases, and as an antiaging and sun proofing agent [15,37,38,39,40,41]. The commercial origin of astaxanthin is from either chemical synthesis or natural resources such as fermentative production (microalgae, yeast) and crustacean byproducts [36,41]. Therefore, more than 95% of the global market refers to synthetically obtained astaxanthin, which presents lower production costs (around $1000/kg) than the biological alternative. Nowadays, the major manufacturers are DSM (The Netherlands), BASF (France), and NHU (China). However, its petrochemical origin limits the final use, which is boosting the natural sources of this carotenoid [14,41]. Thus, astaxanthin from natural sources, including bacteria such as *Paracoccus carotinifaciens*, yeasts like *X. dendrorhous*, or algae like *Haematococcus pluvialis*, is a realistic alternative to synthetic astaxanthin.

The aim of this review is to provide an up-to-date version of the the carotenoids biosynthetic pathway of *X. dendrorhous*, together with the main biotechnology approaches and omics tools that have been applied to this yeast for the improvement of astaxanthin production.

## 2. The Biosynthetic Pathway of Astaxanthin in *X. dendrorhous*

The astaxanthin biosynthetic pathway is encoded by six genes (*crtI*, *crtL*, *crtR*, *crtO*, *crtW*, and *crtZ*), which present an evolutionary pattern (eukaryotic and prokaryotic) characterised by lateral gene transfer and gene duplication events. These genes are more conserved in plants and algae than in any other bacterial phyla, where the structural genes evolve slower than the regulatory genes [44]. This biosynthetic pathway (Figure 1) has been widely studied in *X. dendrorhous* [45,46,47,48]. Thus, the biosynthesis begins with a C5 isoprene unit to which prenyl transferases sequentially add three other isoprenic units [49], resulting in the formation of C20 geranylgeranyl-pyrophosphate (GGPP). The active forms of the isoprene unit are isopentenyl-pyrophosphate (IPP) and its allylic isomer dimethylallyl-pyrophosphate (DMAPP). In most eukaryotes, IPP derives from the mevalonate pathway [50], while in prokaryotes and in plant plastids, it is synthesised via the 2-C-methyl-d-erythritol-4-phosphate (MEP) pathway, which is also known as the non-mevalonate pathway [51].

The *idi* gene encodes IPP isomerase, which catalyses the isomerisation of IPP to DMAPP [52], and then both molecules are joined together, generating C10-geranyl pyrophosphate (GPP), the precursor of monoterpenes [53]. The addition of a second molecule of IPP to GPP by prenyl transferases gives the C15 precursor of sesquiterpenes, farnesyl pyrophosphate (FPP), which is converted into GGPP [54] by the further addition of IPP by the GGPP synthase (encoded by the *crtE* gene). Next, phytoene synthase (encoded by the *crtYB* gene) links two molecules of GGPP in a tail-to-tail manner, yielding phytoene [50,55]. This is the first carotenoid synthesised in the pathway, which is colourless as it has a symmetrical carotenoid skeleton with only three conjugated double bonds. The structural diversity of carotenoids is generated by further modifications, including desaturations, cyclisations, isomerisations, and oxygenations [56]. The phytoene synthase of fungi is a bifunctional enzyme that has both phytoene synthase and lycopene cyclase activities, which gives rise to β-carotene. In this particular enzyme, encoded by the *crtYB* gene, the phytoene synthase and lycopene cyclase activities are located in the C-terminal and N-terminal functional domains, respectively.

Next, phytoene is desaturated by phytoene desaturase (encoded by the *crtI* gene) [3,57] by the incorporation of two, three, four, or five double bonds, thus producing the coloured carotenoids: (i) ζ-carotene (yellow, synthesised by some plants, cyanobacteria, and algae); (ii) neurosporene (yellow, accumulates in *Rhodobacter capsulatus* and *R. sphaeroides*); (iii) lycopene (red, found in most eubacteria and fungi) or (iv) 3,4-didehydrolycopene (found in the mold *Neurospora crassa*) [50], respectively. 

Although there are acyclic carotenoids, the cyclisation of lycopene is a frequent step in the biosynthesis of carotenoids, forming three types of ionone rings: β-, ε-, and γ(gamma)-rings (Britton 1998). The β-ring is the most common, the ε-ring is found in plants and in some algae, and the γ-ring is the rarest one. The lycopene cyclase (also encoded by the *crtYB* gene) [58] sequentially converts the ψ acyclic ends of lycopene to β rings to form γ-carotene and β-carotene [55].

The synthesis of xanthophylls involves the oxidation of post-phytoene carotenoid molecules, mainly from α- and β-carotenes, resulting in oxygenated products with hydroxyl-, epoxy-, and oxo-functional groups. The formation of astaxanthin from β-carotene involves the introduction of a hydroxyl and a keto group at C3 and C4, respectively, for each of the β-ionone rings. Two enzymatic activities convert β-carotene into astaxanthin through several biosynthetic intermediates: a ketolase, which incorporates two 4-keto groups in the molecule of β-carotene, and a hydroxylase, which introduces two 3-hydroxy groups. In *X. dendrorhous*, both activities are included in a single enzyme (astaxanthin synthetase; CrtS), belonging to the cytochrome P450 family and encoded by the *crtS* gene, which sequentially catalyses 4-ketolation of β-carotene followed by 3-hydroxylation [25,26]. In contrast, two independent genes have been described in other astaxanthin producing microorganisms.

A cytochrome P450 reductase, encoded by the *crtR* gene, has been shown to have an auxiliary role to CrtS in *X. dendrorhous*, providing the necessary electrons for substrate oxygenation [59]. A mutant of *X. dendrorhous* lacking the *crtR* gene accumulates β-carotene and is unable to synthesise astaxanthin, demonstrating that CrtR is essential for the synthesis of astaxanthin [59]. In *Saccharomyces cerevisiae* strains expressing *X. dendrorhous* carotenogenic genes, astaxanthin production was only achieved when CrtS was co-expressed with CrtR [60].

The existence of a monocyclic pathway diverging from the dicyclic pathway at neurosporene and proceeding through β-zeacarotene, 3,4-didehydrolycopene, torulene, 3-hydroxy-3′,4′-didehydro-β, ψ-carotene-4-one (HDCO) to the end product 3,3′-dihydroxy-β, ψ-carotene-4,4′-dione (DCD) (Figure 1) was also proposed [42].

## 3. Biotechnology-Based Improvement of Astaxanthin Production in *X. dendrorhous*

Chemical synthesis is relatively complex even for simple carotenoids such as β-carotene. Thus, several companies and academic laboratories have investigated biological sources of astaxanthin. Among these sources, *P. rhodozyma*/*X. dendrorhous* and the microalga *H. pluvialis* stand out because of their ability to synthesise astaxanthin. However, biological production of astaxanthin in natural isolates presents a well-known drawback; the very low specific production. Thus, the specific astaxanthin production of wild type strains of *X. dendrorhous* is 200–400 μg/g of yeast dry weight [61,62]. Besides, the thickness of yeast cell walls and capsule, which apparently hinders astaxanthin uptake, seems to be also relevant for production and downstream processing. In order to obtain a competitive natural source of this xanthophyll it has been necessary to increase this production by a factor of 10–50. Classical random mutagenesis methods by using physical or chemicals agents have been successfully applied [34,43,63,64,65]. Some of these mutants obtained by random mutagenesis and screening with relevant dry cell weight productivity rates [*P. rhodozyma* E5042 (2.5 mg/g); *X. dendrorhous* VKPM Y2476 (4.1 mg/g); *P. rhodozyma* JMU-MVPl14 (6.01 mg/g)] [66,67,68]. Nevertheless, one of the drawbacks of this approach is the introduction of secondary mutations that may affect the physiology, viability, or metabolic capacity of the cell. Besides, genetic instability of the mutants, reduction in the production of biomass, and accumulation of undesired intermediaries have frequently been detected [64,69]. The selection of astaxanthin super-producer mutants in solid medium conditioned the detection of strains due to the low levels of aeration, which inhibit the production of oxygenated carotenoids. As a result, different selection procedures have been developed. On the one hand, positive selection by means of inhibitors of carotenogenesis (β-ionone, diphenylamine) has been developed [70]. On the other hand, negative selection, such as the increased sensitivity of carotenoid-producing strains to antimycin A (a respiratory inhibitor), has been another option [71,72].

The yield improvement of microbial products has been traditionally faced up by means of: (i) treatment with mutagenic agents (e.g., nitrogen mustards, ultraviolet irradiation, X-ray); (ii) increasing the biosynthetic genes; or (iii) redirecting the metabolic precursors [73]. As a consequence, a promising strategy is metabolic engineering (see Table 1), which includes: (i) the overexpression of carotenoid biosynthetic genes (e.g., *crtYB* gene); (ii) the metabolic flow increase towards the synthesis of specific pathway precursor molecules (e.g., geranylgeranyl-pyrophosphate synthase encoding gene (*crtE*)), or (iii) the supplementation with carotenogenesis precursors (e.g., mevalonate) [20,47,62,74,75]. However, this metabolic redirection, which can be included in the new trends of synthetic biology, has needed several methodological updates prior to validating the final effects along the astaxanthin production improvement history. As an example, transformation methods for *Xanthophyllomyces* have been developed and optimised using (i) constitutive promoters from the yeast itself [e.g., actin, glyceraldehyde-3-phosphate dehydrogenase (*gpd*) or NADP-dependent glutamate dehydrogenase (*gdhA*) genes]; (ii) stable integration in the genome by means of the ribosomal DNA of *X. dendrorhous* introduced into the transformation vectors; and (iii) optimised resistance genes (e.g., kanamycin resistance gene of transposon Tn5 or hygromycin resistance gene (*hph*)) [21,76]. Under optimum conditions, the transformation efficiency obtained was 1 × 10^3^ transformants per microgram of plasmid DNA [76].

The carotenoid pathway of *X. dendrorhous* is well established (Figure 1). This fact has enabled the cloning process of all genes involved in phytoene synthesis, phytoene desaturation, and cyclisation and formation of 3-hydroxy and 4-keto. Thus, knowledge of the molecular biology of *X. dendrorhous* allows researchers to direct the genetic modifications and increase the metabolic precursors flow to the carotenoid biosynthetic pathway [48,59,79,80]. In order to divert metabolite flow from the sterol pathway towards carotenoid biosynthesis, *X. dendrorhous* was transformed with the *crtE* cDNA (geranylgeranyl pyrophosphate synthase). Transformants were obtained with higher carotenoid levels including astaxanthin due to increased levels of geranylgeranyl pyrophosphate synthase [74].

The genetic improvement of *Xanthophyllomyces* strains by metabolic engineering has been achieved by upregulating phytoene synthase and lycopene cyclase leading to an increase in β-carotene and echinenone titres. These results suggested that the oxygenation reactions might be rate limiting [48]. The combination of chemical mutagenesis and genetic engineering by targeting limiting reactions (including overexpression of *crtYB* and *crtS* genes) led to the generation of an overproducing strain of astaxanthin (9.7 mg per gram of dry weight) [75]. These authors described the construction of new transformation plasmids for the stepwise expression of the bottleneck genes in the carotenoid biosynthetic pathway. As a result, titres were comparable to those provided by *H. pluvialis*, the leading commercial producer of natural astaxanthin [22].

Enhancement of *crtS* gene transcriptional levels led to an increase in the transcription levels of related genes (*crtE*, *crtYB*, *crtI*) in the astaxanthin biosynthetic pathway. A scheme of carotenoid biosynthesis in *X. dendrorhous* involving alternative bicyclic and monocyclic pathways was proposed by Chi and co-workers in 2015 [81].

The limiting step in the carotenoid pathway is phytoene synthase. In order to increase the *crtYB* gene copy number, integration plasmids were constructed. Thus, the transformants with higher copy number accumulated carotenoid intermediates missed in the parental strain. Some of them are substrates and intermediates of astaxanthin synthase, which can be transformed to astaxanthin by the addition of the astaxanthin synthase gene [82].

Recently, a system to completely delete target diploid genes in *X. dendrorhous* was developed. Diploid *CYP61* genes involved in the synthesis of ergosterol that inhibits the pathway for mevalonate (substrate for isoprenoid biosynthesis), were deleted. Ergosterol biosynthesis was decreased, whereas astaxanthin production was approximately 1.4-fold higher than the parental strain [77].

Many reports have been published about astaxanthin fermentation in *X. dendrorhous*, but there are few reports about large-scale production. In order to produce astaxanthin by biotechnological processes, the scale-up step from lab scale to industry scale is essential. Zheng and co-workers in 2006 [83] reported data from fermentation at 10 m^3^ scale, where the cellular astaxanthin titres reached 2.57 mg/g dry cell.

Light is capital in astaxanthin biosynthesis. Light exposure stimulates total production of carotenoids (mainly astaxanthin) in *Xanthophyllomyces* and has a negative effect on growth [72]. Therefore, the effect of white and ultraviolet light has been tested. Astaxanthin production by fermentation of *X. dendrorhous* was significantly improved at the flask scale by white light (4.0 mg/g) and by ultraviolet light (4.4 mg/g). A semi-industrial process at the 800-L scale for astaxanthin production by fermentation of *X. dendrorhous* was significantly improved by white light and glucose feeding (4.1 mg/g) [84].

## 4. Omics of *X. dendrorhous*: Genomics, Transcriptomics, Proteomics, and Metabolomics

As was indicated above, the metabolic redirection is a suitable reality. It can be enhanced when it is supported by omics technologies, which have been tackled in this yeast from three points of view: genomics, transcriptomics, and proteomics.

The first genomic analysis was done by using pulsed field gel electrophoresis, which defined a putative genome size of 25 Mb [85]. This genome size has been recently redefined by means of the Illumina sequencing methodology (see Table 2 for extraction protocols) [19,86]. As a result, *X. dendrorhous* presents a 19.50 Mb genome with 6385 protein-coding genes [19]. These data were slightly lower (ca. 19 Mb and 6000 ORFs) when two different strains (CBS 7918^T^, CRUB 1149), in addition to the previously sequenced one (CBS 6938), were analysed. Thus, based on these three strains, which can be considered as different varieties in future, the existence of genetic heterogeneity within this red-pigmented yeast was demonstrated [87]. Genome-based pathway analysis presented sterols and carotenoids biosynthesis as the two prominent terpenoid pathways in *X. dendrorhous*. These genes do not follow the typical cluster arrangement for other specific biosynthesis pathways of fungi, which is a peculiarity of this yeast. Besides, the key regulators that control the ethanol accumulation from glucose even under aerobic conditions prior to be re-used on the stationary phase, have been provided from the genome sequence. Those are capital to reach an optimal astaxanthin production increasing the precursors availability by means of pathway engineering [19,87]. Also described was how *X. dendrorhous* is genetically fully equipped to cope with environmental oxidative stress that contributes to its genome shape [88].

The genome sequence also eased the phylogenetic analyses of this astaxanthin-producer basal agaricomycete with uncertain taxonomic placement, which presented *Wallemia* as the most basal agaricomycotinous lineage followed by Tremellomycetes. Besides, the taxonomic analysis defined a sister-group relationship between the core Tremellomycetes and the Cystofilobasidiales [19].

Two sampling points (18 and 72 h) and two different carbon sources (glucose and succinate) were used by Baeza and co-workers [86] to obtain RNA after mechanical rupture by glass beads and Tri-Reagent (Ambion, Foster City, CA, USA) extraction (Table 2). The subsequent RNAseq analysis was the base to determine sequence lengths, expression levels, GC% content, as well as the codon usage and codon context biases of the open reading frames (ORFs) [86]. A total of 1695 ORFs were the basis of the analysis in contrast with one previously described by Verdoes and van Ooyen [89] based on 10 ribosomal genes. Thus, a codon usage table of highly expressed ORFs of *X. dendrorhous* was properly defined, which is highly relevant for the heterologous expression of recombinant genes in the biotechnology industry [90].

Proteome analysis has been the most used omics methodology applied to *X. dendrorhous*, which goes from a reference map and mutant analysis to the comparison in the use of different carbon sources [91,92,93,94]. Besides, the sampling point and procedure help to discriminate those biomolecules directly involved in the process from those contaminants that include noise in the system. For example, this is the case of extracellular proteome (secretome) analysis, where proteins obtained by degradation (degradome) should be avoided [95]. To date, just the intra-cellular proteome has been analysed in *X. dendrorhous.* Those methodologies used for protein extraction, visualisation, and analysis are summarised in Table 2.

Firstly, Martínez-Moya and co-workers [93] developed a heterologous protein identification approach due to the poor genome characterisation at that time. Three time points were selected (lag, late exponential, and stationary growth phases) in minimal media (MM-glucose), where pigment accumulation is evident during the stationary phase. These authors identified two groups of up-regulated proteins: (i) carbohydrate and lipid metabolism proteins, which guarantee acetyl-CoA availability; and (ii) redox-specific proteins (e.g., monooxygenase or cytochrome P450). Both groups presented a scenario that supply the necessary redox potential for the late reactions of the astaxanthin synthesis. These results strongly suggested that astaxanthin production under aerobic conditions is a metabolic tool to scavenge ROS (reactive oxygen species) elements generated as metabolic byproducts in *X. dendrorhous* [93].

The same authors also studied by proteomics and metabolomics the influence of two different carbon sources (glucose (fermentable) or succinate (non-fermentable)) on the metabolism. Lipid and carbohydrate metabolism, carotenogenesis, as well as redox and stress responses proteins, were identified. These data confirmed the connection between the astaxanthin accumulation and oxidative stress in this yeast. Besides, the increase in acetyl-CoA availability when succinate is used as a carbon source was described, which could enhance the cellular respiration rate resulting in ROS elements that induces carotenogenesis [92]. 

In addition to the carbon source, the carbon to nitrogen ratio is crucial for microbial carotenoids production. This relation was analysed at fixed nitrogen concentrations by Pan and co-workers [94] in *X. dendrorhous*. Intriguingly, cell growth and astaxanthin accumulation were connected to the increasing of the carbon to nitrogen ratio, whereas the astaxanthin amount per cell was inverse. These metabolic adaptations were studied by two-dimensional electrophoresis analysis, where the up-regulation of redox- and stress-associated proteins, as well as carotenogenesis proteins were observed. In contrast, nine proteins involved in the astaxanthin synthesis were down-regulated and the de novo pigments synthesis was inhibited, which justify this peculiar unbalance between growth and cellular pigment accumulation [94].

As a result of nitrosoguanidine treatments, different coloured mutants (red, orange, pink, yellow, and white), which were analysed under the proteomics methodologies, were obtained by Barbachano-Torres and co-workers [91]. The red mutants were total carotenoids (mainly astaxanthin) overproducers, whereas orange and white ones accumulated phytoene as a result of phytoene dehydrogenase mutation. This analysis also demonstrated the close relation among the tricarboxylic acid cycle, stress response, and the carotenogenic process.

## 5. Conclusions and Future Prospects

Commercial use of astaxanthin in aquiculture, as well as in human nutrition and health is well-defined and growing year after year. Nowadays, astaxanthin production is tackled by chemical synthesis, which allows a profitable cost/benefit ratio. However, its petrochemical origin presents regulatory concerns, which is shifting attention in favour of biological sources such as bacteria, yeasts, or algae. Unfortunately, some methodological (e.g., light induction, product extraction) or biotechnological difficulties (e.g., low production) are the current key concerns of a more eco-friendly production process. The practical advances (culture conditions, transformation processes, high throughput screening systems, genetic and metabolic know-how, scale up processes) supported by omics technologies are boosting the carotenoids production by natural procedures. Thus, *X. dendrorhous*, which has been approved by the FDA for the commercial production of astaxanthin, is a good candidate to allow the natural production of astaxanthin with a proper isomerism and chemical structure. There is good knowledge and a methodological basis, but the natural production of astaxanthin has plenty of room for improvement.

## Figures and Tables

**Table 1:** Examples summary of *X. dendrorhous* (*P. rhodozyma*) genetic engineering.

Targets	Approach	Result	Ref.
*crtYB* gene	Deactivation	No carotenoids	[48]
*crtYB* gene	Overexpression	Accumulation of β-carotene and echinenone	[48]
*crtI* gene	Overexpression	Increase torulene and HDCO and decrease echinenone, l-carotene and astaxanthin	[48]
*crtR* gene	Description of its role	Required together with the *crtS* gene for the conversion of β-carotene to astaxanthin.	[59]
double *cyp61* genes	Deletion	Enhanced astaxanthin production by 1,4-fold compared with the parental strain	[77]
*crtE* gene under Padh4r	Evaluation of promotors	Increase in intracellular astaxanthin by 1.7-fold compared with parental	[78]
*acaT*, *hmgS* and *hmgR* genes	Triple overexpression	Enhanced volumetric astaxanthin production by 1.4-fold compared with that of the control strain	[23]
*acaT/hmgS/hmgR/crtE/crtS* genes	Combined overexpression	Enhanced volumetric astaxanthin production by 2.1-fold higher compared with the control strain	[23]
Combination of conventional mutagenesis and *crtYB* gene expression	Combined overexpression	22 times higher astaxanthin specific production than for the wild type	[75]

**Table 2:** Summary of the biomolecules extraction protocols described for *X. dendrorhous* (*P. rhodozyma*).

Media	Collection	Disruption	Analysis Method	Ref.
**DNA**				
YPD medium. 21 °C, 5 days [96]	Culture: 15 mL Suspended: 0.5 mL YPD	Breaking system: 300 μL of glass beads (0.25–0.5 mm diameter). Swing mill (Retsch MM200) at a frequency of 30/s.	Agarose gel and ethidium bromide stain. Fluorescence densitometry measurement.	[19]
		Sample cleaning: Supernatant collected and purified by phenol/chloroform/isoamyl alcohol extraction. DNA precipitation overnight at −20 °C by 100% ice-cold ethanol (2.5 volume) and 1/10 volume of 3 M sodium acetate solution. 70% ice-cold ethanol. Dry at room temperature. DNA pellet resuspension in 30 μL H_2_O. Store at 4 °C.		
YM medium (100 mL). 22 °C, up to stationary phase	Centrifugation	Breaking system: DNA isolated from protoplasts: 2× wash 50 mM EDTA pH 7.5. Novozyme 234 plus LET buffer (500 mM EDTA, 7.5% 2-mercaptoethanol, 10 mM Tris pH 7.5). 16 h, 37 °C. NDS solution. (2 mg/mL proteinase K in 500 mM EDTA, 1% lauryl sarcosine and 10 mM Tris-HCl, pH 7.5). 24 h, 50 °C.	DNA quantitation: 260/280 ratio (1.7–1.9) and 260/230 ratio (>2) by using a V-630 UV–vis Spectrophotometer.	[85,86]
		Sample cleaning: Phenolic extraction (pH 8.0): 3× wash saturated phenol. 3× phenol: chloroform: isoamyl alcohol (25:24:1). 1× chloroform: isoamyl alcohol (24:1). DNA was precipitated with 98% ethanol and washed with 70% ethanol. Dry DNA resuspended in Tris: EDTA (10:1; pH 8.0) plus 40 μg/mL of RNase A. 37 °C, 30 min. Repeat phenolic extraction		
YM broth (15 mL) at 20 °C, 72 h	Centrifugation	Breaking system: Modified phenol:chloroform:isoamyl alcohol method [97,98]: Resuspend in 500 µL lysis buffer (10 mM Tris-HCl pH 8.0, 100 mM NaCl, 1 mM EDTA (pH 8.0), 2% Triton X-100, 1% SDS). Add an equal volume of phenol/chloroform (1∶1 *v*/*v*). Shake vigorously (Ika-Vibrax VXR shaker) at 1800 rpm, 20 minutes, R/T. Centrifuge at 14,000 rpm, 20 min, 4 °C.	N/A	[88]
		Sample cleaning: Ethanol precipitation.		
**RNA**				
YPD medium. 21 °C, 5 days [96]		NucleoSpin^®^ RNA Plant kit (MACHEREY-NAGEL GmbH & Co. KG) (Following the manufacturer instructions).	RNA quality by using Nano-Photometer (IMPLEN) 1.5% agarose gel and ethidium bromide stain	[19]
Vogel minimal medium (MMv) supplemented with 2% glucose or 2% succinate	Early exponential phase (18 h) Initial stationary phase (72 h)	Breaking system: Mechanical rupture of cell pellets. 0.5 mm glass beads (BioSpec). Vortexing for 10 min. Add Tri-Reagent (Ambion). R/T 10 min.	RNA quantitation: 260/280 ratio (>1.9) by using a V-630 UV–vis Spectrophotometer	[86]
		Sample cleaning: Add 200 μL of chloroform per mL of Tri-Reagent. Mix. Centrifuge: 4000× *g*, 5 min. Recover supernatant. 2× acidic phenol: chloroform (1:1) extractions. Precipitate: 2 volume of isopropanol. 10 min, R/T. 1× wash 75% ethanol. Resuspend in RNase-free water. RNA samples at a 260/280 ratio >1.9, measured using a V-630 UV–vis Spectrophotometer, were used for next-generation sequencing.		
**Proteins**				
Minimal medium plus 2% glucose or succinate as carbon sources [99] Preculture: 10 mL Culture: 250 mL in 1-L flask inoculated with 2.5 mL of seed culture. 22 °C, 120 rpm	Centrifugation: 5000× *g*, 10 min, 4 °C. Pellet washed twice with ice-cold water. Centrifuge: 5000 × *g*, 10 min, 4 °C. Freeze in liquid N_2_. Stored at −80 °C	Breaking system: Lyophilise cells. Add an equal volume (±500 μL) of glass beads (500 μm). Add 500 μL of lysis buffer (100 mM sodium. bicarbonate, pH 8.8, 0.5% Triton × 100, 1 mM phenylmethylsulfonyl fluoride (PMSF) and protease inhibitors). 15 min in on ice. Shake at 30 s at 4.5 m/s (RiboLyzer). Chill on ice, 1 min between shaking steps.	Bidimensional gel (pI range: 3–10 NL, 17 cm strips) Coomassie brilliant blue Trypsin digestion MALDI-TOF-MS identification	[92,93]
		Sample cleaning: Remove cell debris by centrifugation (15,000 rpm, 20 min, 4 °C. 10% *v*/*v* DNase-RNase solution (0.5 M Tris-HCl, pH 7.0, 0.5 M MgCl_2_, 100 μg/mL RNAse and 2 μL DNase). 1 h, 4 °C. Add water up to 2.5 mL plus 200 μL of 0.5 M Tris (pH 6.8) and 20 μL of 1 M dithiothreitol (DTT). R/T. 30 min. 600 μL of water-saturated phenol. R/T. 30 min. Centrifuge: 5000× *g*, 10 min, 4 °C Add to supernatant 20 μL of 1 M DTT and 30 μL of 8 M ammonium acetate. R/T. 30 min. Precipitate. 2 mL of cold (−20 °C) methanol. Centrifuge: 13,000 rpm, 4 °C, 30 min. 2× wash: 70% (*v*/*v*) cold ethanol at −20 °C. Resuspend pellet: 200 μL of buffer (8 M urea, 2 M thiourea, 2% CHAPS, 0.01% [*w*/*v*] bromophenol blue). Store at −80 °C.		
YM medium 20-h old culture (beginning of carotenoid biosynthesis) 20 °C and 150 rpm.	Centrifugation: 5000 rpm, 10 min.	Breaking system: Liquid nitrogen in a mortar Resuspend fine powder in 5 mL of buffer (50 mM Tris–HCl pH 7.4, 0.5 mM PMSF). Add 5 mL of 100 mM sodium carbonate. 1 h on ice. Centrifuge: 6000 rpm, 20 min, 4 °C. Supernatant precipitation: 10% final concentration of trichloroacetic acid (TCA). Centrifuge: 10,000 rpm for 15 min.	Bidimensional gel (pI range: 3–10, 11 cm strips) Colloidal Coomassie [100]	[91]
		Sample cleaning: 1× acetone. 1× 70% ethanol. 200 μL rehydration buffer (7 M urea, 2 M thiourea, 1% CHAPS, 0.5% Triton X-100, 40 mM Tris–HCl, 0.5% ampholytes 3–10, and 0.1% bromophenol blue). Centrifuge: 10,000 rpm, 15 min. Desalt: Micro Bio Spin G-30 columns (Bio-Rad) and rehydration buffer (Bio-Rad, Hercules, CA, USA).		
Seed culture: 20 g/L glucose, 10 g/L yeast extract, 20 g/L peptone. 22 °C, 200 rpm, 48 h. 250-mL flask containing 30 mL Inoculate 3 mL in a 250-mL flask containing 30 mL of seed media. 22 °C, 200 rpm, 24 h Fermentation: (20 g/L glucose, 0.2 g/L yeast extract, 0.5 g/L (NH_4_)_2_SO_4_, 1.0 g/L KH_2_PO_4_, 0.1 g/L NaCl, 0.5 g/L MgSO_4_-7H_2_O, and 0.1 g/L CaCl_2_-2H_2_O). 22 °C, 200 rpm, 144 h	Centrifugation: 8000× *g*, 10 min, 8 °C, 96 h.	Breaking system: Resuspend pellet in deionised water. 2× 30 kpsi high-pressure disrupter (Constant Systems Limited, Northants, UK). 10,000× *g*, 15 min, 4 °C. Collect supernatant 0.5 mg dissolved in 500 µL rehydration buffer [8 M urea, 4% CHAPS, 2% IPG buffer and 40 mM DTT (Dithiothreitol)].	Bidimensional gel (pI range: 4–7) Silver stain Trypsin digestion MALDI-TOF/TOF-MS identification	[94]
